# Hamstrings mechanical properties profiling in football players of different competitive levels and positions after a repeated sprint protocol

**DOI:** 10.3389/fphys.2023.1315564

**Published:** 2024-01-04

**Authors:** Ricardo Pimenta, Hugo Antunes, Paula Bruno, A. P. Veloso

**Affiliations:** ^1^ CIPER, Centro Interdisciplinar de Performance Humana, Faculdade de Motricidade Humana, Universidade de Lisboa, Lisboa, Portugal; ^2^ Research Center of the Polytechnic Institute of Maia (N2i), Maia Polytechnic Institute (IPMAIA), Maia, Portugal; ^3^ Futebol Clube Famalicão—Futebol SAD, Department of Rehabilitation and Performance, Famalicão, Portugal

**Keywords:** biceps femoris long head, semitendinosus, shear wave elastography, shear modulus, soccer

## Abstract

**Purpose:** This study compares the average speed, knee flexor peak torque and shear modulus of the hamstrings after a repeated sprint task, in football players of different competitive levels and playing positions.

**Methods:** Fifty-four football field players without hamstring strain injury history participated, 15 being categorized as professional (2nd league) and 39 as semi-professional (17 in 3rd and 22 in 4th league). Muscle shear modulus was assessed using ultrasound-based shear wave elastography at rest and at 20% of maximal voluntary isometric effort before and immediately after the repeated sprint protocol.

**Results:** No significant differences were seen in average sprint speed between competitive levels (*p* = 0.07; η2*p* = 0.28) and positions (*p* = 0.052; η2*p* = 0.29). Moreover, the sprint fatigue index showed no significant differences between competitive levels (*p* = 0.14; η2*p* = 0.08) and playing positions (*p* = 0.89; η2*p* = 0.05). No significant differences were observed in hamstring shear modulus changes between competitive levels (*p* = 0.94; η2*p* = 0.03) and positions (*p* = 0.92; η2*p* = 0.03). Peak torque changes also showed non-significant association with competitive levels (*p* = 0.46; η2*p* = 0.03) and positions (*p* = 0.60; η2*p* = 0.02).

**Conclusion:** The results of this study suggest that the average sprint speed performance parameter and mechanical parameters are not able to distinguish football players of different competitive levels and positions.

## Introduction

The profiling of athletes of different modalities reveals physiological, anthropometric and physical patterns that are useful for the assessment and monitoring of performance parameters by providing a framework that coaches and sports scientists can use to compare athletes within or between teams (Slimani and Nikolaidis, 2019; Tierney et al., 2021). Specifically, in football, the physiological, anthropometric and physical profile has been studied in the past years (Abrantes et al., 2004; Sporis et al., 2009; Lago-Peñas et al., 2011) making it possible to describe and compare physical capacities of players of different positions (Schwesig et al., 2017) and competitive levels (Tierney et al., 2021). However, scientific research still reveals a gap in the knowledge of the profiling of football players, especially when it comes to the characterization of the muscular properties of specific muscles that can significantly influence the players’ running performance such as the hamstrings muscle group.

The hamstring is a biarticular muscle group that acts simultaneously at two joints by participating in knee flexion and hip extension. Moreover, the hamstrings have an important role for the development of horizontal force components during sprinting in non-fatigued conditions (Edouard et al., 2018). In the context of football matches, players have to sprint also with a change of direction component. This requires stabilization of the knee joint, which is achieved through a distinct contribution of the medial and lateral hamstrings (Zebis et al., 2009). Previous studies demonstrated a decrease in maximal force production of the hamstrings immediately post-match (Bueno et al., 2021); however, as peak torque measurements do not provide information about the mechanical contribution of individual muscles, shear wave elastography has been proposed to quantify mechanical muscle properties (i.e., muscle shear modulus). Indeed, changes in torque production are correlated with changes in muscle shear modulus (Bouillard et al., 2012).

To the best of our knowledge, only two studies have analyzed the behavior of the hamstring mechanical tissue properties after repeated sprints: in healthy individuals (Pimenta et al., 2023c) and football players with and without hamstring strain injury (Pimenta et al., 2023b). The former demonstrated a significant increase in the biceps femoris long head (BFlh) contribution without changes in semitendinosus (ST) (Pimenta et al., 2023c), while the latter showed a significant increase in the contribution of BFlh and semimembranosus (SM) (Pimenta et al., 2023b). Considering the larger muscle volumes of ST and BFlh muscles in sprinters compared to non-sprinters (Handsfield et al., 2017), a higher mechanical contribution by ST and BFlh at higher competitive levels can be expected given the higher sprint volume and intensity. It can also be expected that faster players (e.g., forwards) will show a higher ST contribution due to its considerably higher proportion of fast-twitch fibers (Fournier et al., 2022), whereas players covering long distances (e.g., midfielders) show a higher BFlh contribution due to its higher number of type I/IIa fibers (Dahmane et al., 2005). Therefore, a study comprising the effect of repeated sprints on the hamstring mechanical properties in football players of different competitive levels and positions would be of great interest and utility for the sports science and professional community, adding information to the knowledge framework that already exists comprising other significant components of a football player profile.

The present study aims at comparing the effects of a repeated sprint protocol on the sprint performance and hamstrings shear modulus pattern between players with different competitive levels and positions. The hypothesis was i) a higher sprint performance and a higher mechanical contribution of the ST and BFlh at higher competitive levels; ii) a higher sprint performance and higher mechanical contribution of ST for forwards when compared to midfielders and defenders; iii) a higher mechanical contribution of BFlh for midfielders compared to forwards and defenders.

## Methods

Ten clubs were invited to participate in the present study, resulting in 54 football field players (age: 23.5 ± 3.6 years; height: 178.3 ± 6.4 cm; body mass: 73.2 ± 7.1 kg): 15 categorized as professional (2nd league) and 39 as semi-professional (17 on 3rd league and 22 on 4th league) according to the official Portuguese league website. The sample size in relation to the field position was composed by 23 defenders, 15 midfielders and 16 forwards. All participants read and signed an informed consent form prior to participating in the study. The Ethical Committee at the Faculty of Human Kinetics at the University of Lisbon approved the study (#5/2021). Participants were instructed to avoid any strenuous activities 24 h before the test to minimize confounding factors. The exclusion criteria were replicated from previous study (Pimenta et al., 2023b), alongside exclusion of previous hamstring injury.

### Dynamometry

The knee flexor torque was measured at a sampling rate of 1,000 Hz using custom-made equipment (Pimenta et al., 2023b). Participants were placed in the prone position, with the hips in neutral anatomical position, knees flexed at 30° (0° = full extension) as previously reported (Pimenta et al., 2023b). Both feet were fixed in a foot holder containing a force transducer (Model STC, Vishay Precision, Malvern, PA, United States) at the heel level to collect the linear force perpendicular to the leg orientation and with the ankle at 90°. Force data were amplified (Model UA73.202, Sensor Techniques, Cowbridge, UK), digitally converted (USB-230 Series, Measurement Computing Corporation Norton, MA, United States), recorded using the DAQami software (v4.1, Measurement Computing Corporation, Norton, MA, United States), and multiplied by the perpendicular distance between the force transducer center and the femoral lateral condyle in order to estimate the knee torque. Visual feedback of force production was provided to individuals during the assessments.

### Sprint performance

Sprint performance was evaluated by a 10 × 30 m repeated sprint protocol using a two-point stance, with participants positioned 1 m behind the photocells. The average sprint speed was recorded using four photocells and data was processed by the Chronojump software (version: 2.1.1-16, Chronojump Boscosystem, Barcelona, Spain).

### Shear wave elastography

Hamstrings shear modulus was assessed using two similar ultrasound scanners (Aixplorer, v11; Supersonic Imagine, Aix-en-Provence, France; Aixplorer, v12; Supersonic Imagine, Aix-en-Provence, France) in shear wave elastography (SWE) mode (musculoskeletal preset, penetrate mode, smoothing level 5, opacity 100%, scale: 0–800 kPa for active (i.e., during contraction), coupled with a linear transducer array (SL10-2, 2–10 MHz. Super Linear, Vermon, Tours, France). The SWE procedures were detailed in the previous study with a similar test protocol (Pimenta et al., 2023b). The transducer was placed to align with the muscle fascicles orientation, and to perform minimal pressure during the measurements. To maximize the window of opportunity of the effects in both tasks, both examiners collected data simultaneously in the pairs BFlh + SM and ST + BFsh. The utilization of casts and the measurement with two examiners was previously validated (Pimenta et al., 2023d).

### Protocol

Participants visited the Centro de Alto Rendimento Jamor indoors facility, where wind and temperature had no effect on sprint performance and shear modulus assessment, respectively. Testing started by assessing the passive shear modulus for each muscle. For this purpose, two videoclips of 20-s were recorded. Then, individuals were asked to perform 10 submaximal knee flexions at a self-perceived low intensity to prepare and familiarize with the equipment for the maximum voluntary isometric contraction (MVIC) evaluation, which consisted of two 3-s trials with 30-s of recovery between trials. Based on the highest PT on the tested limb, individuals familiarized themselves with the 20% of MVIC through trials using visual feedback. Subsequently, the active shear modulus was then measured twice for each muscle at 20% of MVIC, each trial lasting ∼30-s. After active shear modulus measures, a standardized warm-up protocol for sprinting was performed (Pimenta et al., 2023b). Immediately after the warm-up, a 10 × 30-m repeated sprint task with 30-s interval between repetitions was performed. Then, post-task active shear modulus measurements were conducted followed by two MVIC trials. The order of the measurements in each muscle was randomized.

### Data processing

Shear wave elastography data were processed using automated MATLAB routines (The Mathworks Inc., Natick, MA, United States) (Mendes et al., 2020). For the shear modulus calculation, each clip exported from Aixplorer’s software was sequenced in. jpeg images. Image processing converted each pixel of the color map into a value of the Young’s modulus based on the recorded color scale. The largest region of interest in the elastogram window was determined by avoiding aponeuroses and tissue artifacts (e.g., vessels), and the values were averaged to obtain a representative muscle value. Within each trial, the most stable Young’s modulus values over ∼20-s in the active condition were averaged and divided by 3 to better represent the muscle shear elastic modulus (Bercoff et al., 2004). The shear modulus of each muscle was considered for analysis.

### Statistical analysis

Data analysis was performed using IBM SPSS Statistics 27.0 (IBM Corporation, Armonk, NY, United States). To assess the effect of fatigue in each muscle a delta (Δ) active shear modulus (post-pre) was calculated for each muscle (Pimenta et al., 2023a). A one-way between-groups MANOVA was performed to investigate competitive level differences in active shear modulus. A one-way between-groups MANOVA was performed to investigate field positions differences in active shear modulus. For both MANOVA’s, a preliminary assumption testing was conducted to check for normality, linearity, univariate and multivariate outliers, homogeneity of variance-covariance matrices, and multicollinearity, with no serious violations noted. The Fatigue index for sprint performance was calculated using the following equation:

Fatigueindex=slowestsprint‐fastestsprint/fastestsprint

The fatigue index comparison was examined using a one-way ANOVA for competitive level and playing position. Peak torque changes was calculated using the following equation:

Peak Torque changes=(post−protocol peak torque)‐(pre−protocol peak torque)/(pre−protocol peak torque)

A one-way ANOVA was used to analyze the peak torque differences between competitive levels and playing positions. The partial eta square (η2p) values were reported as a measure of the effect size of the MANOVA’s and ANOVA findings, classified as small (η2*p* = 0.01–0.05), medium (η2*p* = 0.06–0.013), and large (η2*p* > 0.14) effects (Cohen, 2013). Significance was set at *p* < 0.05.

## Results

**FIGURE 1:**
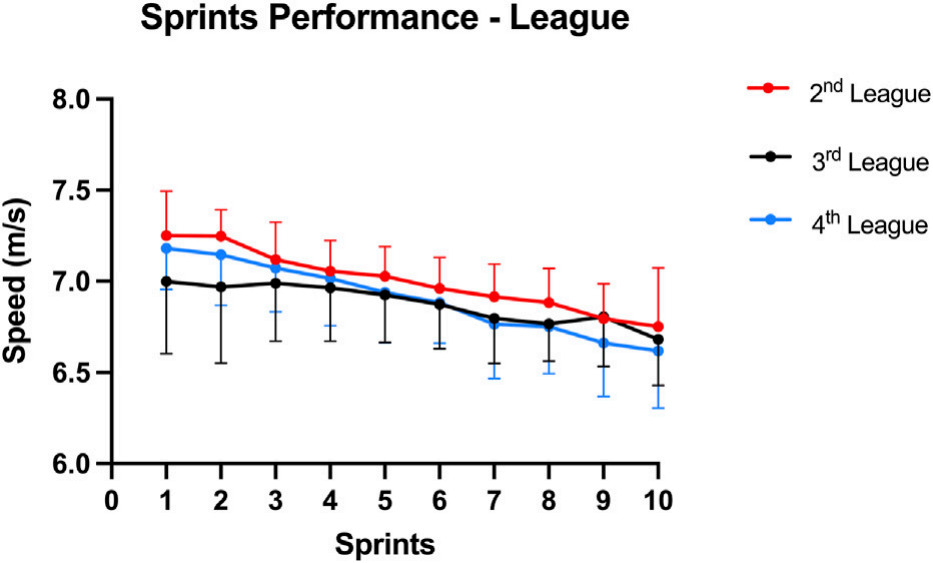
Average sprint speed (m/s) during the repeated sprinting task, in players of 2nd league (red), 3rd league (black) and 4th league (blue). Error bars represent the standard deviation.

**FIGURE 2:**
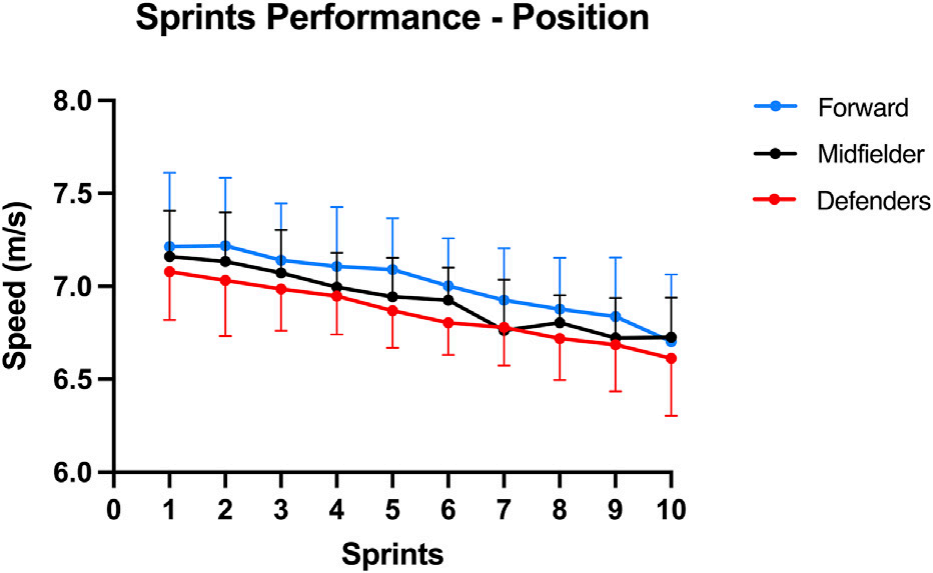
Average sprint speed (m/s) during the repeated sprint task, comparison between playing positions: in forwards (blue), in midfielders (black) and defenders (red). Error bars represent the standard deviation.

Regarding the sprints performance between competitive levels (Figure 1) no significant differences were seen, *F* (20, 82) = 1.61; *p* = 0.07; Wilks’ Λ = 0.52; η2p = 0.28. In relation to the sprints performance by position (Figure 2) no significant differences were detected, *F* (20, 82) = 1.69; *p* = 0.052; Wilks’ Λ = 0.50; η2p = 0.29.

**FIGURE 3:**
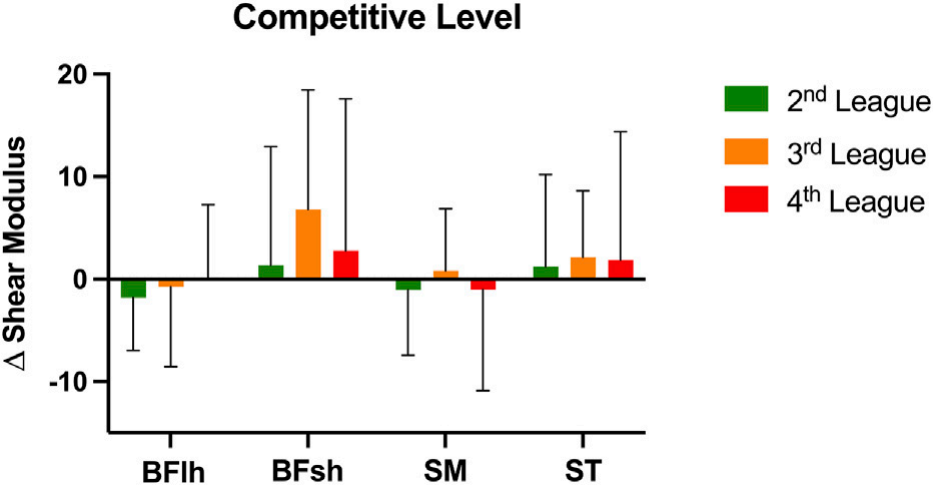
Delta (Δ) shear modulus variation (post—pre) of all hamstring muscles: Biceps Femoris long head (BFlh), Biceps Femoris short head (BFsh), Semimembranosus (SM), and Semitendinosus (ST), in each competitive level: Second League (green), Third League (orange), Fourth League (red). Bars represent mean values and error bars represent standard deviation.

**FIGURE 4:**
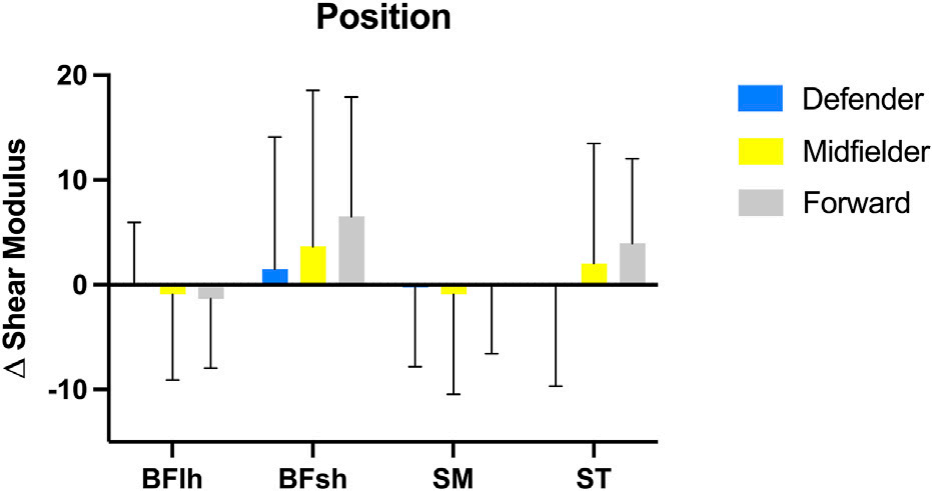
Delta (Δ) shear modulus variation (post—pre) of all hamstring muscles: Biceps Femoris long head (BFlh), Biceps Femoris short head (BFsh), Semimembranosus (SM), and Semitendinosus (ST) in each competitive field position: Defender (blue), Midfielder (yellow), Forward (gray). Bars represent mean values and error bars represent standard deviation.

The Δ shear modulus analysis of all hamstring muscles by competition level (Figure 3) showed no significant differences between competitive levels on the combined dependent variables, *F* (8, 96) = 0.36; *p* = 0.94; Wilks’ Λ = 0.94; η2p = 0.03. Figure 4 presents the Δ shear modulus of all hamstring muscles by position on the field with no significant differences on the combined dependent variables, *F* (8, 96) = 0.40; *p* = 0.92; Wilks’ Λ = 0.94; η2p = 0.03.

Fatigue index comparisons between competitive levels (2nd league: −6.76% ± 5.68%; 3rd league: −4.25% ± 6.48%; 4th league: −7.78% ± 4.35%; *p* = 0.14; η2p = 0.08) and playing positions (defenders: −6.46% ± 5.79%; midfielders: −5.99% ± 4.02%; forwards: −6.85% ± 5.54%; *p* = 0.89; η2p = 0.05).

Peak torque differences comparisons revealed no significant differences between competitive levels (2nd league: −7.8% ± 0.96%; 3rd league: −5.8% ± 0.86%; 4th league: −8.3% ± 0.91%; *p* = 0.46; η2p = 0.03) and playing positions (defenders: −6.7% ± 0.85%; midfielders: −8.8% ± 0.93%; forwards: −5.6% ± 0.91%; *p* = 0.60; η2p = 0.02).

## Discussion

To the best of our knowledge, this is the first study that compares the effects of a repeated sprint protocol on the sprint performance and hamstrings mechanical properties between players with different competitive levels and positions. The main findings were no differences in the sprint performance and hamstring shear modulus between competitive levels and positions. Since in our study no differences were found between competitive levels it was possible to perform a comparison between field positions using players across all competitive levels.

A higher average sprint speed was seen in 2nd league compared to 3rd and 4th league players (Figure 1); however, contrary to our initial hypothesis no significant differences were seen between the competitive levels. These results are in contrast to previous literature also assessing portuguese players, Abrantes et al. (2004) having reported significant differences in the mean sprint time using the Bangsbo repeated-sprint ability (RSA) protocol (Bangsbo, 1994) between professional football players, with players from 1st national division being significantly faster than 2nd league players. These professional players displayed significant differences in relation to semi-professionals (1st regional) (Abrantes et al., 2004). However, the present study did not include first league players and thus a comparison between professionals was not possible. Moreover, the lowest division in our study (4th league) was from the first national league which differs from a regional championship. Contrary to our protocol, the Bangsbo RSA includes a change of direction component and it has been shown that shuttle-type RSA protocols are associated with slower repetition times and a reduced sprint decrement (Thurlow et al., 2023).

Regarding the comparison between positions (Figure 2), the forwards showed a higher sprint speed, followed by midfielders and lastly defenders; however, also contrary to our hypothesis no significant differences were found between the positions. This is in accordance with previous work using the Bangsbo protocol (Bangsbo, 1994) that did not find any statistical differences with respect to best time and average time (Kaplan, 2010). Furthermore, Brahim et al. (2016) showed that forwards performed better in mixed-direction repeated sprint tests, midfielders were better in mixed-direction repeated sprint tests that involved a longer linear sprinting distance (6 × 40 m (20 + 20 m)), and defenders had better scores in linear repeated sprint tests such as our protocol. It should be noted that the above mentioned protocols cannot be compared with the present protocol as it was designed to induce fatigue on the hamstrings muscles instead of being designed according to the sprinting skills of the player positions. A similar linear repeated sprint protocol (7 × 30-m) was employed with recreational players with the results also showing no significant differences in sprint performance between players of different positions (Lockie et al., 2019). Since linear and multidirectional sprint tasks represent independent skills expression (Salaj and Markovic, 2011), it is possible that while players might present different performances in multidirectional sprint protocols, they may present no significant differences in linear sprints performance as in the present results.

The fatigue index formula used in this study was also applied in a previous study (Morcillo et al., 2015) showing a 7.10% decrease after a similar RSA protocol in professional players. The inability to maintain sprint speed in subsequent repetitions is also proposed to be affected by maximal aerobic capacity (Girard et al., 2011) which can distinguish elite from non-elite football players. In fact, higher fatigue levels were verified in amateur players (4th league) compared to semi-professional players, although non-significant. Importantly, this maximal aerobic capacity can also be influenced by the player’s position (Slimani and Nikolaidis, 2019). In relation to the peak torque analysis, no significant differences were seen between competitive levels and playing positions. Using a different formula to calculate peak torque changes upon a fatiguing sprint task, Lord et al. (2018) found a −6% change for the dominant leg, a value closer to those reported in this study (Lord et al., 2018). No significant differences in peak torque changes were observed between the different field positions, contrary to previous work (Cometti et al., 2001; Śliwowski et al., 2017); given the lack of investigation regarding this topic, future studies should replicate a similar protocol comparing a different sample of football players (e.g., elite football players vs. sub-elite or amateur) to potentially enable a better explanation of these results.

Regarding the mechanical hamstring tissue properties (analyzed as Δ shear modulus) and contrary to our initial hypothesis no significant differences were found across leagues and positions. According to the mechanical tension experienced by the BFlh along the sprint cycle (Thelen et al., 2005) and the effects of an eccentric task (Goreau et al., 2022), an increase in BFlh shear modulus would have been expected. Indeed, previous studies using the same repeated sprint protocol showed a significant increase in the BFlh (Pimenta et al., 2023b; 2023c). Despite the disparity in the results, it is worth mentioning that the samples of these previous studies consisted of healthy individuals (Pimenta et al., 2023c) and of football players with hamstring strain injuries (Pimenta et al., 2023b). Healthy individuals (non-football players) may present different muscle volume configurations compared to football players, this being related to the muscle adaptations according to the sport stimulus (Krustrup et al., 2010). Supporting this assumption, Handsfield et al. (2017) reported that sprinters had considerably larger ST, BFlh and SM volumes compared to the non-sprinter sample (Handsfield et al., 2017). Nevertheless, as football players from higher competitive levels are likely submitted to higher sprinting action demands resulting in superior hamstring muscle adaptations than players in lower competitive levels, it is curious that this was not apparent in the present study.

Apart from the competitive level, the playing position has a significant effect on the game actions of a football player. Research has been consistent demonstrating that certain positions require more explosive-type efforts while others are submitted to more resistant-type efforts (Di Salvo et al., 2013). In a recent review, authors noted that male football players with a higher proportion of type II muscle fibers had faster 30 m sprint times and achieved a greater total sprinting distance, and that more type II muscle fibers and a higher muscle volume in rugby players were strong determinants of maximal muscle power in sprinting (Hopwood et al., 2023). Hence, it would be logical that players in a field position requiring explosive actions presented larger and higher proportions of type II muscle fibers and consequently a higher shear modulus change in a muscle with a type II muscle fiber phenotype.

The present study demonstrated a higher speed (Figure 1) and positive change in the ST Δ shear modulus of forwards (Figure 4), although no significant differences were found. Having a type II phenotype (Fournier et al., 2022) and being a fusiform muscle (Azizi and Deslauriers, 2014), the ST geometry and composition is well suited for explosive tasks but not as resistant as other hamstring muscles (Kellis, 2018; Fournier et al., 2022). As football sport demands vary between player positions, it would be expected that faster players could present a higher relative ST mechanical contribution and more resistant players a higher relative BFlh mechanical contribution, as BFlh has a more balanced type muscle fiber composition compared to ST (Kellis, 2018; Fournier et al., 2022). Importantly, the competitive level between professional football leagues can have an impact on the data interpretation, such as analyzing data from the Premier League (Di Salvo et al., 2013) which is more competitive than the 2^nd^ league of the present study. For instance, there is evidence that players from top-level European leagues cover 10% of total distance at high intensity (Carling, 2011; Andrzejewski et al., 2015) compared to the 6.4% from the Croatian league (Modric et al., 2019). As such, one could expect a lower proportion of high-intensity activity (including sprints) in players from the 2nd, 3rd and 4th division. Overall, the present study indicates that a repeated sprint protocol 10 × 30 m with 30 s of rest induce fatigue in both professional and semi-professional players and between positions on the neuromuscular parameters. However, it was not possible to distinguish the response in hamstring mechanical properties and neuromuscular parameters across the assessed competitive levels. One possible reason could be in part attributed to the progress of available knowledge in strength and conditioning applications in football and its interpretation by club sports scientists. This warrants further investigation.

This study has some limitations. Firstly, shear moduli measurements were limited to one site per muscle to minimize the time required for data collection and thus allow accurate examination of the acute fatigue effects. Therefore, the present findings are based on the assumption that the effects were site-independent. Secondly, the present study examined the hamstring mechanical properties in isometric contractions after repeated sprints, whereas the magnitude of the effects would be greater during the sprints themselves as eccentric contractions are more demanding. However, to this date, the low sampling rate of this methodology prevents such measurements. Competitive level and playing position data might be significantly influenced by the football leagues analyzed due to different intensity and technical levels.

## Conclusion

The results of the present study indicated that it was not possible to distinguish football players from different competitive levels and positions when analyzing the sprint performance, hamstring shear modulus and peak torque after a repeated sprint task. However, it should be noted that in the present study, the tasks were analytical, therefore the results can be task-specific since game contexts varied in terms of competitive levels and positions. These results may also suggest that the disparities in physical characteristics between competitive levels and positions have significantly diminished. This could be attributed to specific training characteristics and a heightened influence of physical attributes in football. We encourage further studies to analyze this hypothesis.

## Data Availability

The raw data supporting the conclusion of this article will be made available by the authors, without undue reservation.
